# A Cardiac Time Bomb: Stroke as the Initial Presentation of a Large Atrial Myxoma

**DOI:** 10.7759/cureus.113109

**Published:** 2026-07-21

**Authors:** Sudarsh TS, Swathi GR, S Sureya Vayshnava Kumar, Yogesh Subramanian, Roshni Subramaniyan, Karthigeyan TS, Sahasyaa Adalarasan, Jayaprakash N, Hariharan C

**Affiliations:** 1 Internal Medicine, Madras Medical College, Chennai, IND; 2 Internal Medicine, Rajiv Gandhi Government General Hospital, Chennai, IND; 3 Internal Medicine, Madras Medical College and Rajiv Gandhi Government General Hospital, Chennai, IND; 4 Medicine, Madras Medical College, Chennai, IND

**Keywords:** atrial myxoma, ischemic stroke, rare, stroke, tumor, young

## Abstract

Primary cardiac tumors are rare. Atrial myxomas are one of the most frequent benign tumors of the heart. These tumors typically manifest as obstructive symptoms, including dyspnea and syncope. Constitutional symptoms, including fever, weight loss, and fatigue, are also common. Embolic phenomena are rare. Neurological manifestations primarily occur as ischemic infarction, whereas hemorrhagic transformation is infrequent. We present a young adult who experienced an ischemic stroke followed by hemorrhagic transformation, ultimately diagnosed with an atrial myxoma. During the patient's hospitalization, neurological improvement was noted while he received antiplatelet and statin therapy. The definitive treatment, consisting of surgical excision, was performed in this patient, followed by echocardiographic surveillance to monitor for recurrence.

## Introduction

Atrial myxoma is the most frequently encountered primary benign neoplasm of the heart [1]. Approximately three-quarters of these tumors arise in the left atrium, typically originating near the fossa ovalis of the interatrial septum or the mitral valve annulus. About 20% are found in the right atrium, while biatrial involvement occurs in nearly 5% of patients [2-5]. Owing to their mobile nature, cardiac myxomas carry a significant risk of embolic events. Their clinical presentation is highly variable and may include positional or exertional dyspnea, syncope, ischemic stroke, peripheral embolization, fever, weight loss, and constitutional symptoms resulting from cytokine production [3]. Among the neurological complications associated with atrial myxoma, ischemic stroke is the most common [6]. Transthoracic echocardiography is generally the first-line imaging technique for diagnosis, whereas transesophageal echocardiography offers better visualization of tumor morphology, attachment site, and the presence of additional intracardiac masses. Surgical resection remains the treatment of choice and should be performed promptly to prevent embolic complications, sudden circulatory obstruction, and tumor recurrence [3].

Ischemic stroke, which accounts for the majority of stroke cases, may result from thrombotic or embolic vascular occlusion [7]. In patients with atrial myxoma, ischemic stroke most commonly occurs through a cardioembolic mechanism, either due to embolization of tumor fragments or thrombi formed on the tumor surface, leading to cerebral arterial occlusion [7].

Here, we describe a young male who presented with an acute ischemic stroke complicated by hemorrhagic transformation and was later found to have a large left atrial myxoma. This case underscores the need to actively consider cardiac causes in young patients presenting with stroke.

## Case presentation

A 20-year-old South Asian male patient presented to the emergency department with a sudden onset of dizziness and weakness affecting his right upper and lower limbs while playing football. He had no prior history of hypertension, diabetes, cardiac disease, or any other conventional vascular risk factors.

On general examination, he was afebrile, with a blood pressure of 110/70 mmHg and a heart rate of 102 beats per minute. Peripheral pulses were well felt. Cardiovascular examination revealed normal heart sounds without any audible murmurs. Abdominal and other systemic examinations were unremarkable, and there were no external features suggestive of congenital disease.

Neurologically, the patient was conscious and able to follow commands. There was a deviation of the angle of the mouth to the left. Muscle tone was reduced on the right side, with power graded as 2/5 in the right upper limb and 3/5 in the right lower limb. The plantar reflex was extensor on the right side.

**Figure 1:**
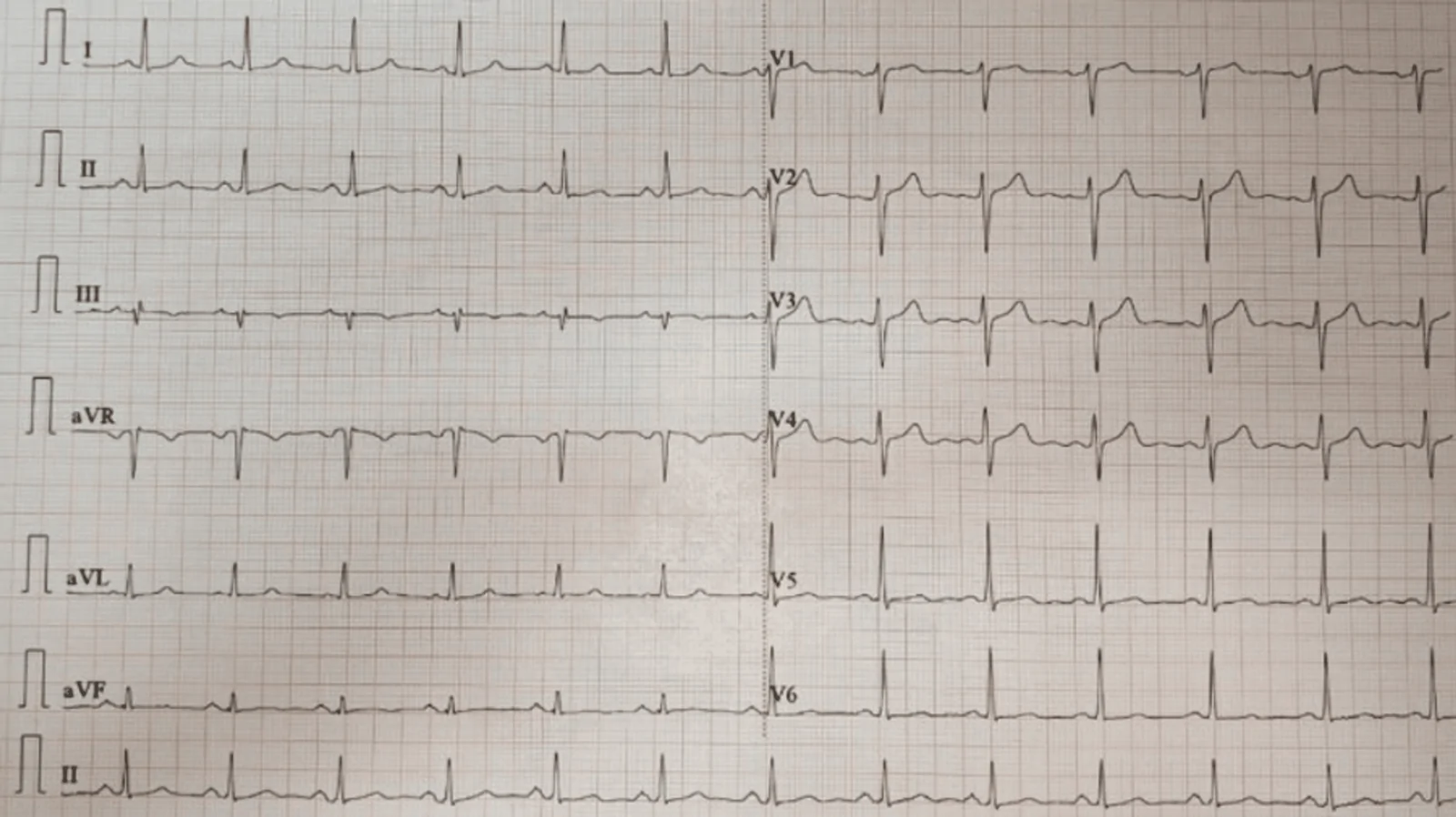
Electrocardiography of the patient showing normal sinus rhythm.

Electrocardiography showed a normal sinus rhythm with a normal axis and regular RR intervals (Figure 1).

**Figure 2:**
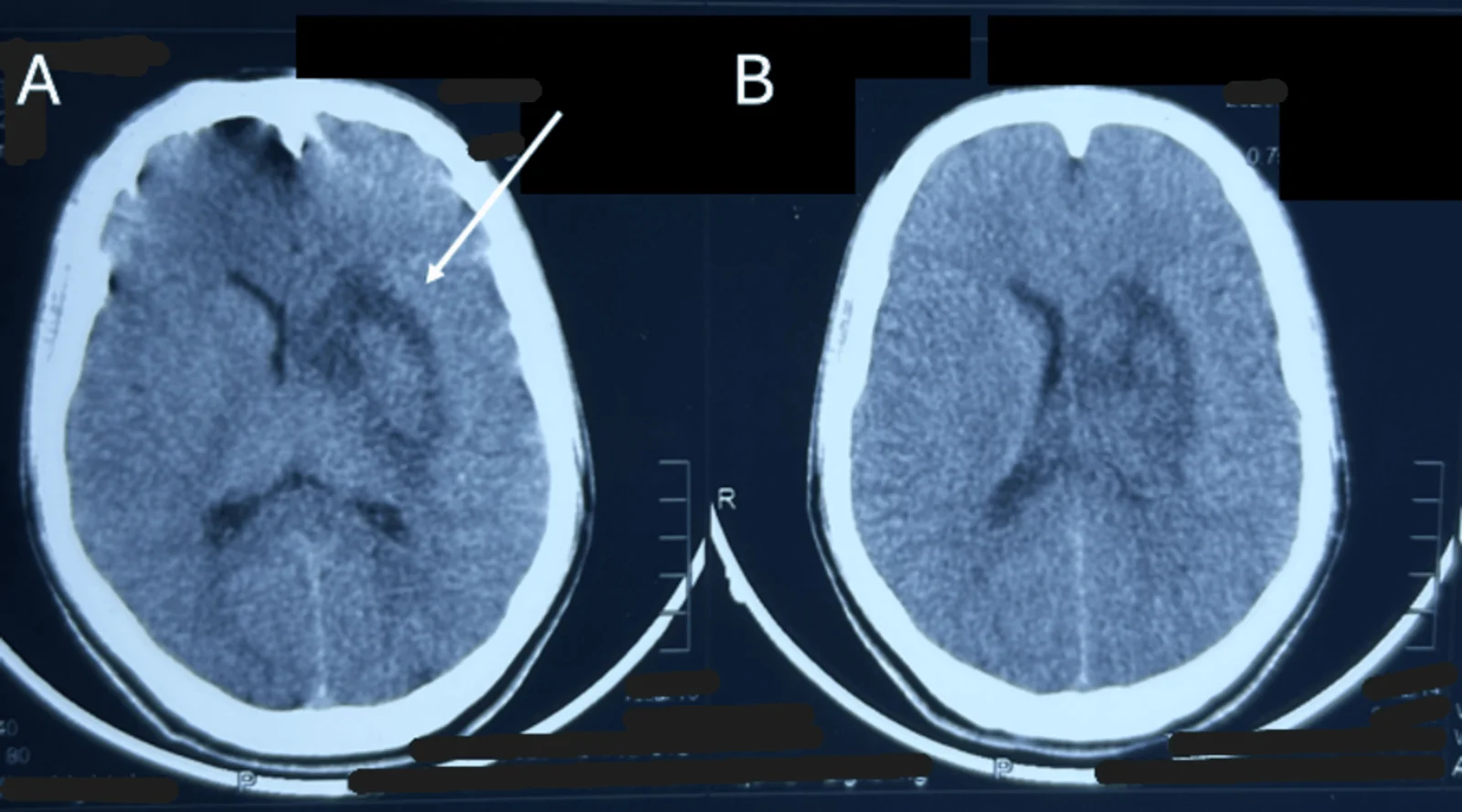
CT scan of the head showed hypodensity in the left temporoparietal region with HU 60, indicating early hemorrhagic transformation, likely an acute infarct. HU, Hounsfield Unit.

Neuroimaging was performed for further evaluation. A non-contrast CT scan of the brain showed hemorrhagic transformation within the infarcted area, without any significant mass effect or midline shift (Figure 2A, 2B).

**Figure 3:**
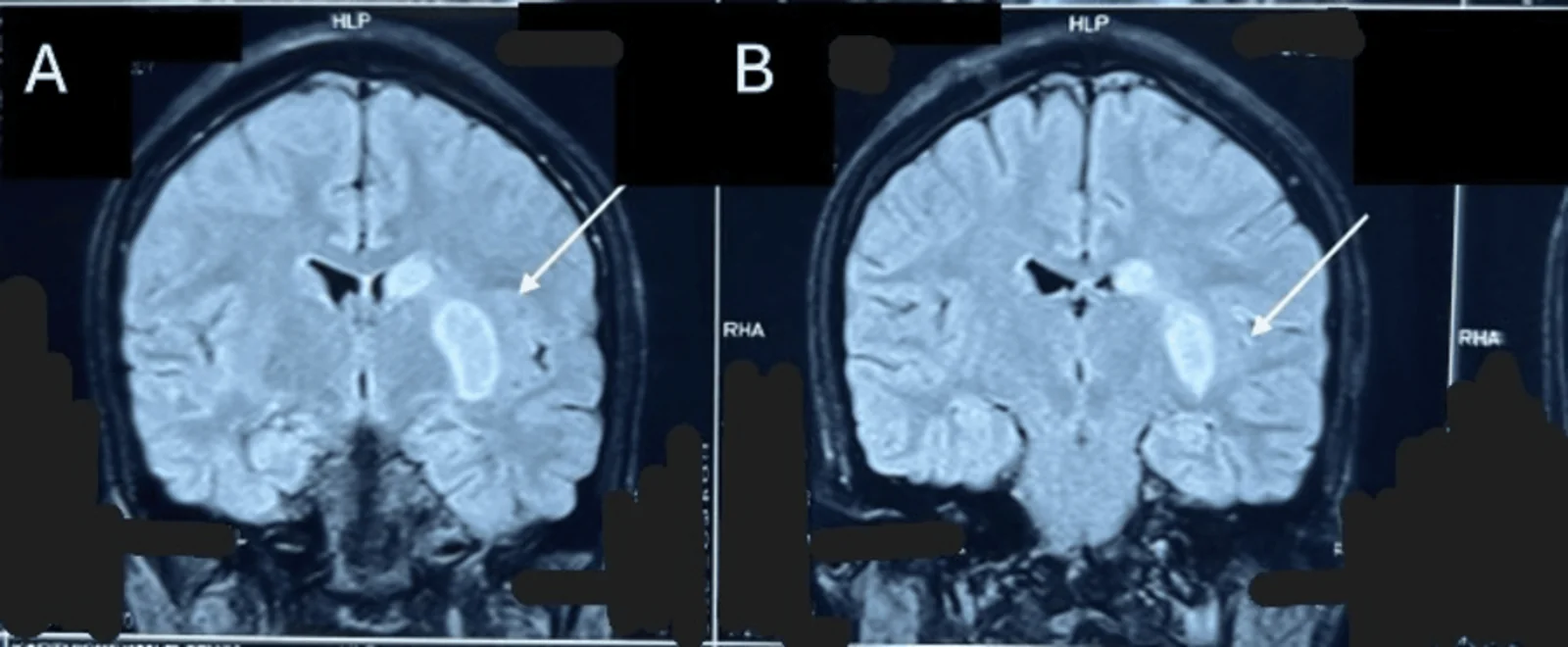
MRI brain showing large area of diffusion restriction within blooming within noted in left ganglio-capsular region suggestive of acute infarcts with hemorrhagic changes.

Subsequent MRI of the brain demonstrated an acute ischemic infarct in the capsuloganglionic region, with diffusion restriction on diffusion-weighted imaging and surrounding signal heterogeneity, suggestive of early hemorrhagic transformation (Figure 3A, 3B).

**Figure 4:**
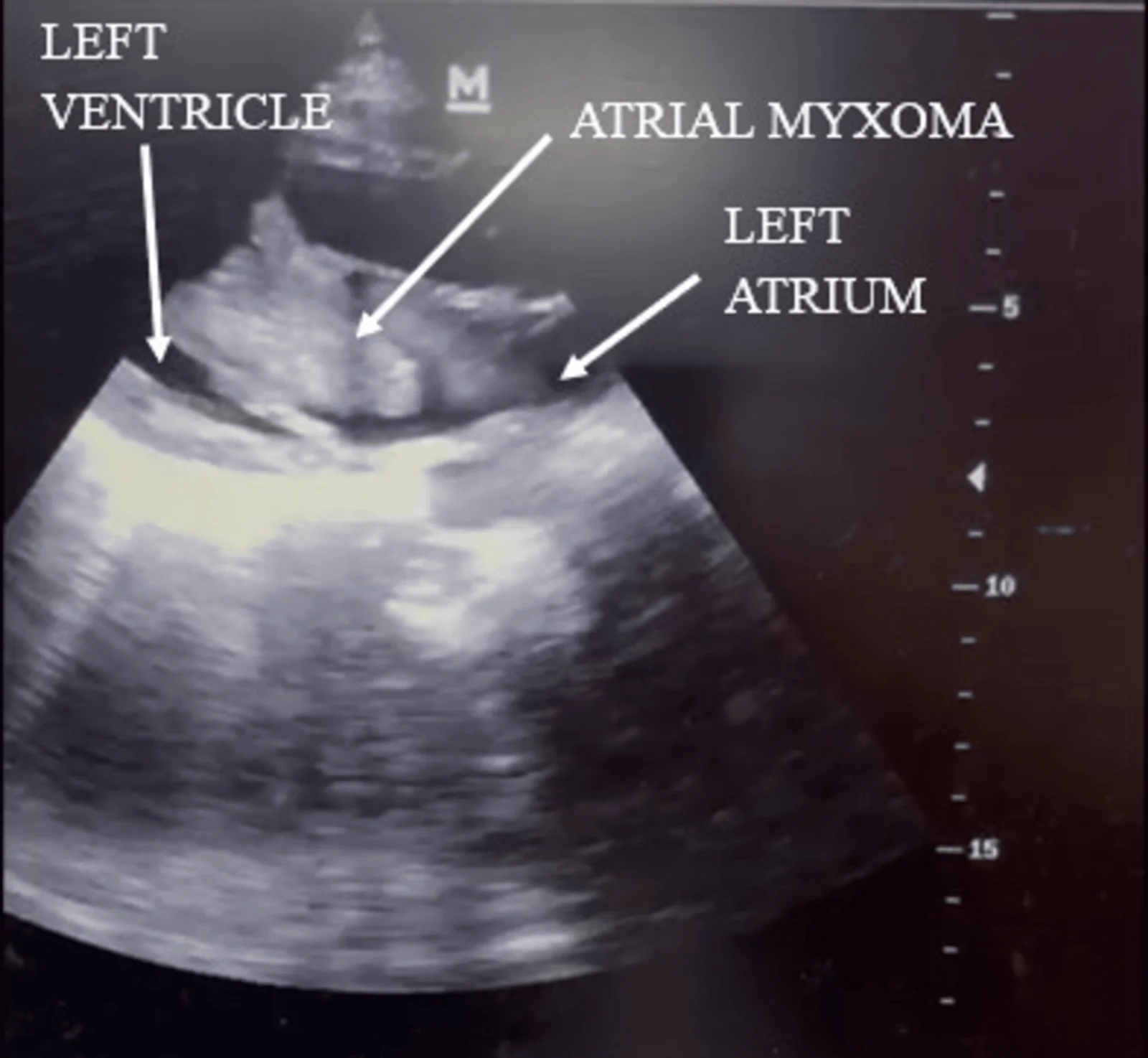
Echocardiography, which revealed an 82*40 mm large echogenic heterogeneous mass in left atrium attached to the interatrial septum, which was mobile and protruding into the left ventricle. The appearance was suggestive of a left atrial myxoma.

Transthoracic echocardiography revealed a mobile mass in the left atrium attached to the interatrial septum, consistent with a diagnosis of left atrial myxoma (Figure 4).

**Figure 5:**
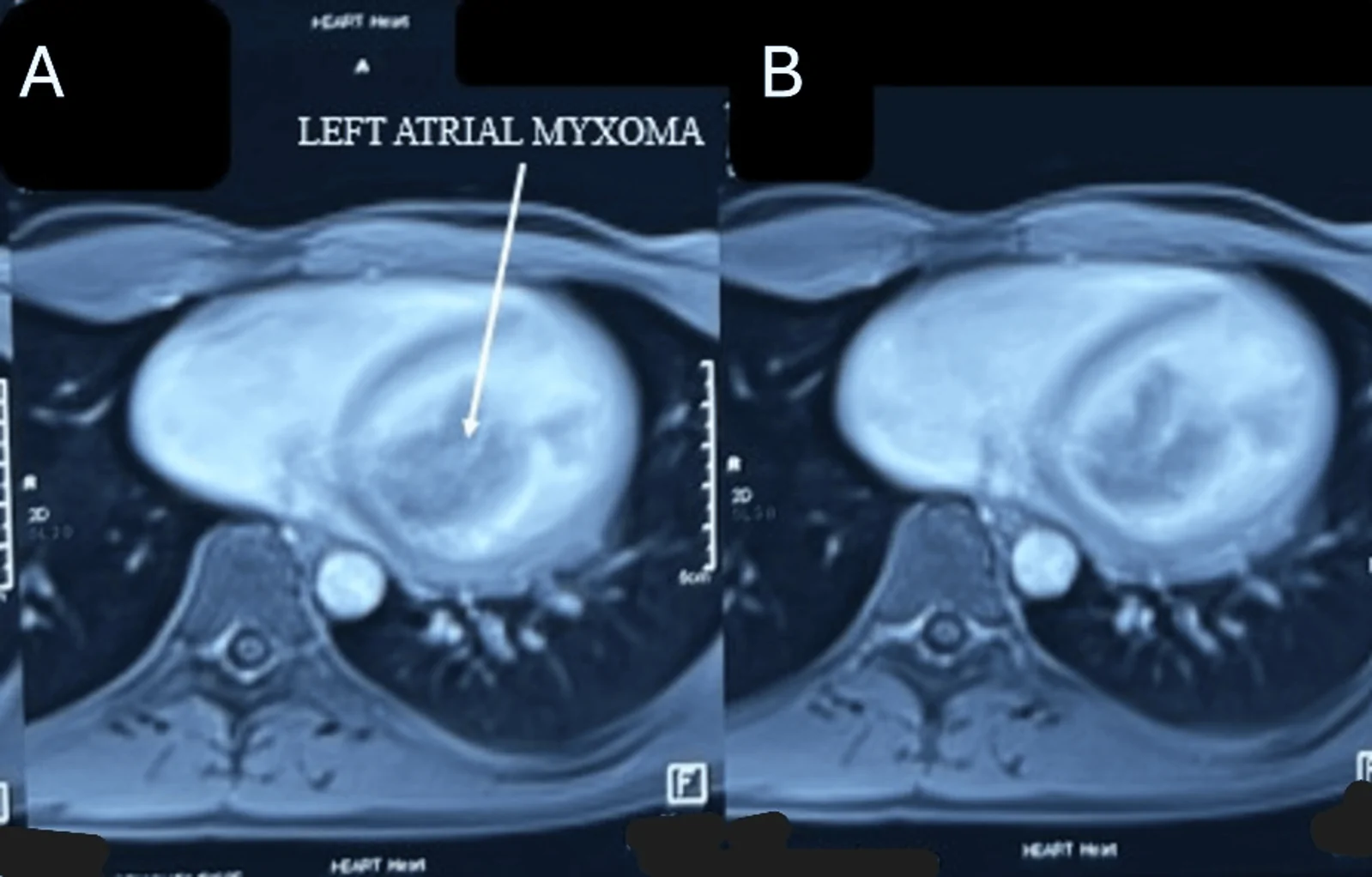
Cardiac MRI, which demonstrated a large heterogeneous lesion in the left atrium.

A cardiac MRI was performed, which revealed a relatively large hypointense heterogeneous irregular lesion in the left atrium and attached to the atrial wall, prolapsing into the mitral valve on cine images with heterogeneous post-contrast enhancement (Figure 5A, 5B).

**Figure 6:**
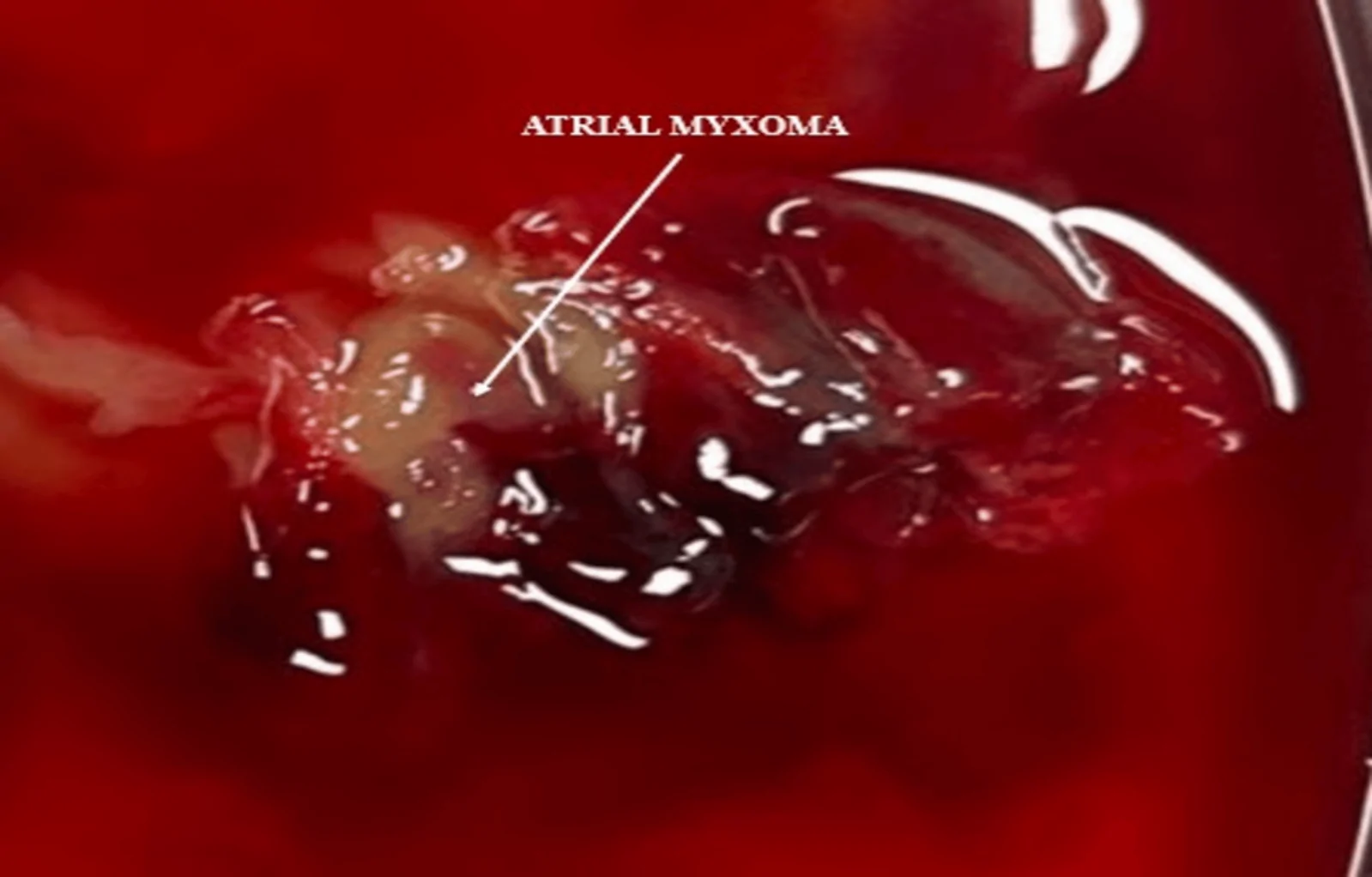
Surgical specimen of the left atrial mass consistent with a large friable tumor.

The patient was started on aspirin and high-intensity statin therapy as secondary prevention while awaiting surgery [8,9]. He clinically improved even before undergoing cardiac surgery, with a Glasgow Coma Scale score of 15/15 and full motor strength (Medical Research Council grade 5/5 [10]) in the right upper and lower limbs. This explains the likelihood of thrombus emboli along with the tumor. In view of a cardioembolic stroke, he underwent surgical resection of the tumor (Figure 6). The postoperative period was uneventful.

## Discussion

Primary cardiac tumors are rare, with an incidence between 0.0017 and 0.19%. Atrial myxomas constitute nearly half of the benign tumors of the heart [1]. These tumors usually arise from the interatrial septum [2]. Their clinical presentation depends largely on tumor size, location, and mobility, with obstructive features often mimicking functional mitral stenosis. Constitutional symptoms, including fever, weight loss, and fatigue, are the next most common. Embolic phenomena are the least common. In the present case, the patient presented with a cardioembolic stroke as the initial manifestation, emphasizing the importance of evaluating for a cardiac source in patients with stroke, particularly in the absence of conventional vascular risk factors [11].

Although atrial myxoma is benign, it can result in serious complications due to its propensity to embolise. The neurological manifestations of atrial myxoma are most commonly due to ischemic stroke [6]. Myxomas that are smooth are often larger and present with obstructive symptoms, while villous and friable myxomas are the ones that are responsible for embolic phenomena [8]. Embolization does not occur only from the migration of fragments from the larger tumor but also from the adherent thrombus on the surface of the tumor that dislodges along with it [9]. In our patient, the observed clinical improvement following antiplatelet therapy prior to surgical intervention may suggest a possible thromboembolic contribution; however, this observation should be interpreted as a clinical inference rather than definitive evidence that embolization resulted predominantly from thrombus rather than tumor tissue alone.

In this case, more than the size, the mobility of the tumor likely increased its embolic potential [12]. Hemorrhagic transformation in the setting of myxoma-related stroke is relatively uncommon. The underlying mechanism is likely multifactorial, involving disruption of the blood-brain barrier, vascular injury caused by tumor emboli, and reperfusion-related changes, rendering the infarcted brain tissue more susceptible to secondary bleeding [13].

Compared with previously published reports, our case demonstrated both shared and unique characteristics. The patient was notably young and did not present the conventional cardiovascular risk factors, supporting observations that cardioembolic events associated with myxoma frequently occur in individuals without typical stroke risk factors [2,14].

A multicenter case-control study conducted by Qiao et al. evaluated 402 patients, of whom 54 had cardiac myxoma-associated ischemic stroke (CM-IS), and 348 had cardiac myxoma without evidence of stroke [15]. During the acute phase, the median National Institutes of Health Stroke Scale (NIHSS) score [16]was 3 (interquartile range: 0-10), and affected individuals commonly presented with a mixture of neurological, cardiac, and constitutional manifestations. Multivariate analysis demonstrated that several factors were independently linked to an increased risk of CM-IS, including tumor width less than 30 mm (OR = 2.652, 95% CI: 1.061-6.627, P = 0.037), marked tumor mobility (OR = 2.700, 95% CI: 1.357-5.371, P = 0.005), thrombus formation on the tumor surface (OR = 1.856, 95% CI: 1.003-3.434, P = 0.049), and lower B-type natriuretic peptide (BNP) concentrations (OR = 0.995, 95% CI: 0.989-0.999, P = 0.047). Among patients with CM-IS who underwent surgical excision, the estimated survival rate at three years was 95.7% (95% CI: 94.9-96.5) [15].

A review conducted by Ekinci & Donnan examined six cases managed at their institution along with 107 cases identified from the published literature [6]. The analysis demonstrated a higher prevalence among females compared with males, with a female-to-male ratio of 3:2. Although neurological manifestations varied considerably and often overlapped, ischemic stroke was the most frequent presentation, occurring in 83% of patients and involving multiple vascular territories in 41% of cases. Other reported clinical features included syncope (28%), psychiatric symptoms (23%), headache (15%), and seizures (12%). Echocardiography was the diagnostic modality most commonly used to establish the diagnosis [6].

Chen et al. reported a rare neurological complication of cardiac myxoma in the form of subarachnoid hemorrhage (SAH) [17]. Previous reports have generally attributed myxoma-associated SAH to the rupture of myxomatous intracerebral aneurysms. However, their study described an unusual case of angiographically negative SAH in a young patient with a left atrial myxoma. This finding suggests that mechanisms other than the rupture of myxomatous intracerebral aneurysms may contribute to the development of SAH in patients with cardiac myxoma [17].

Cases of atrial myxoma in pediatric patients have also been documented. Their clinical presentation resembles that seen in adults, commonly including hemiparesis or hemiplegia along with additional neurological manifestations such as dysarthria, aphasia, or altered mental status. In all reported cases, the diagnosis of cardiac myxoma was established following the occurrence of stroke [18]. A subset of patients was identified as having Carney complex, an autosomal dominant multiple endocrine neoplasia syndrome that is commonly associated with mutations in the PRKAR1A gene [19]. Heart myxomas are the most common noncutaneous manifestation in Carney's complex [18-20]. In these affected individuals, these tumors may arise at any age, with a median age of detection around 20 years. They are often multifocal, may involve any cardiac chamber, and are associated with a high risk of recurrence [18].

This case highlights the importance of considering less common causes of stroke, particularly cardiac sources, in young patients without traditional vascular risk factors.

Imaging plays a pivotal role in establishing the diagnosis. Transthoracic echocardiography is typically the first-line modality for detecting intracardiac masses and assessing their mobility and site of attachment [21]. Cardiac MRI provides superior tissue characterization and helps differentiate tumors from thrombus [22], while CT imaging offers additional anatomical detail useful for surgical planning. Early utilization of these imaging modalities enables prompt diagnosis and appropriate management. Management is initially supportive, especially in the presence of hemorrhagic transformation. Perioperative anticoagulation therapy has been shown to be highly beneficial in patients with stroke secondary to cardiac myxoma who undergo thrombolysis, in those with a low post-thrombectomy bleeding risk, and in patients who have not yet experienced embolic events. Definitive management is subsequently achieved through surgical excision of the tumor [23].

Definitive treatment of atrial myxoma is surgical excision, which generally yields excellent outcomes with a low recurrence rate in sporadic cases. Early surgical intervention is essential to reduce the risk of further embolic events [24]. Regular follow-up with periodic echocardiographic surveillance is essential for early detection of tumor recurrence [25].

Although early surgical removal of cardiac myxoma is generally advised to minimize the risk of life-threatening systemic embolization, existing guidelines do not provide clear recommendations regarding the optimal timing of surgery or the use of preoperative anticoagulant and antithrombotic therapy. This is largely due to the limited number of reported cases of atrial myxoma-associated acute ischemic stroke (AIS). Studies have demonstrated that a longer delay between the onset of stroke and subsequent tumor resection is significantly associated with an increased risk of recurrent stroke [26,27].

Surgical resection remains the definitive treatment for cardiac myxomas, with median sternotomy continuing to be the most widely used operative approach. However, robotic-assisted cardiac surgery has emerged as an important advancement and a viable alternative for myxoma excision. This technique provides improved visualization and better exposure of the tumor, facilitating more effective resection. Furthermore, multi-articulating robotic instruments offer greater dexterity and precision than the long-shafted instruments typically used in conventional minimally invasive cardiac procedures [28,29]. Despite being performed within a confined operative field, robotic myxoma excision allows enhanced manoeuvrability and superior visualization of intracardiac structures through the use of high-magnification optical systems [29,30].

One limitation of this case report is that the histopathological details of the excised tumor and long-term postoperative follow-up data were not available, which limits assessment of recurrence risk and long-term clinical outcomes.

## Conclusions

Ischemic stroke is the most common neurological presentation of atrial myxoma, while hemorrhagic transformation in this setting is relatively rare. Embolic events can occur either from fragmentation of the tumour itself or from thrombus formation on its surface. Our patient, who presented with an ischemic stroke, underwent transthoracic echocardiography, from which a cardiac myxoma was diagnosed. Preoperatively, he was managed with antiplatelet treatment along with high-dose statin therapy. Finally, surgical excision was done to remove the tumor.

Recognising an underlying cardiac source early, especially in young patients without typical vascular risk factors, is essential, as it allows for timely surgical treatment and helps prevent recurrent embolic episodes.

## References

[1] Reynen K (1995). Cardiac myxomas. N Engl J Med.

[2] Pinede L, Duhaut P, Loire R (2001). Clinical presentation of left atrial cardiac myxoma. A series of 112 consecutive cases. Medicine (Baltimore).

[3] Vaidya Y, Sharma S (2026). Atrial myxoma. StatPearls.

[4] Jelic J, Milicić D, Alfirević I (1996). Cardiac myxoma: diagnostic approach, surgical treatment and follow-up. A twenty years experience. J Cardiovasc Surg (Torino).

[5] Li H, Guo H, Xiong H, Xu J, Wang W, Hu S (2016). Clinical features and surgical results of right atrial myxoma. J Card Surg.

[6] Ekinci EI, Donnan GA (2004). Neurological manifestations of cardiac myxoma: a review of the literature and report of cases. Intern Med J.

[7] Lui F, Khan Suheb MZ, Patti L (2026). Ischemic stroke. StatPearls.

[8] Hasan M, Abdelmaseih R, Faluk M, Chacko J, Nasser H (2020). Atrial myxoma, a rare cause of sudden cardiac death: a case report and review of literature. Cureus.

[9] Gil J, Marmelo B, Abreu L, Antunes H, Dos Santos LF, Cabral JC (2017). Silent cardiac tumor with neurological manifestations. J Cardiol Cases.

[10] Naqvi U, Margetis K, Sherman AL (2026). Muscle strength grading. StatPearls.

[11] Alrushaid S, Salmeen A (2025). Cardioembolic stroke secondary to a cardiac myxoma. Radiol Case Rep.

[12] Lee VH, Connolly HM, Brown RD Jr (2007). Central nervous system manifestations of cardiac myxoma. Arch Neurol.

[13] Hong JM, Kim DS, Kim M (2021). Hemorrhagic transformation after ischemic stroke: mechanisms and management. Front Neurol.

[14] Touilite M, Ait Ami S, Chraa M, Louhab N (2026). Cardiac myxoma presenting with multiple ischemic strokes and critical limb ischemia: a case report. Cureus.

[15] Qiao ML, Ma L, Wang CB (2023). Clinical features, risk factors and survival in cardiac myxoma-related ischemic stroke: a multicenter case-control study. J Neurol Sci.

[16] (2026). NIH stroke scale. https://www.ninds.nih.gov/health-information/stroke/assess-and-treat/nih-stroke-scale.

[17] Chen C, Xu Y, Zhuang W, Zhao Z, Wang Y (2021). Subarachnoid hemorrhage following ischemic stroke caused by atrial myxoma. Cureus.

[18] Tona C, Nosadini M, Pelizza MF (2020). Cardiac myxoma as a rare cause of pediatric arterial ischemic stroke: case report and literature review. Neuropediatrics.

[19] Kamilaris CD, Faucz FR, Voutetakis A, Stratakis CA (2019). Carney complex. Exp Clin Endocrinol Diabetes.

[20] Canali E, Serani M, Tarzia P, Ciampi P, Canestrelli S, Calò L (2023). Echocardiography in cardioembolic stroke prevention. Eur Heart J Suppl.

[21] Stratakis CA, Kirschner LS, Carney JA (2001). Clinical and molecular features of the Carney complex: diagnostic criteria and recommendations for patient evaluation. J Clin Endocrinol Metab.

[22] Motwani M, Kidambi A, Herzog BA, Uddin A, Greenwood JP, Plein S (2013). MR imaging of cardiac tumors and masses: a review of methods and clinical applications. Radiology.

[23] Chen Y, Huang Q, Bai C, Zhang H, Zhang B (2024). Preoperative management and anticoagulant efficacy in atrial myxoma-associated acute ischemic stroke: a case report and literature review. Front Cardiovasc Med.

[24] Keeling IM, Oberwalder P, Anelli-Monti M (2002). Cardiac myxomas: 24 years of experience in 49 patients. Eur J Cardiothorac Surg.

[25] Kuroczyński W, Peivandi AA, Ewald P, Pruefer D, Heinemann M, Vahl CF (2009). Cardiac myxomas: short- and long-term follow-up. Cardiol J.

[26] Wu X, Chen T, Han Y, Wang K, Zhou J (2024). Left atrial myxoma as a rare cause of stroke. Heliyon.

[27] Rosário M, Fonseca AC, Sotero FD, Ferro JM (2019). Neurological complications of cardiac tumors. Curr Neurol Neurosci Rep.

[28] Deitz RL, Ogami T, Ashraf SF, Kaczorowski DJ, Bonatti J (2022). Technical aspects of robotically assisted left atrial myxoma resection. Ann Cardiothorac Surg.

[29] Mendyka D, Płonek T, Jędrasek T (2024). The therapeutic potential of different surgical approaches in the management of cardiac myxoma: a systematic review. J Clin Med.

[30] Hassan M, Smith JM (2012). Robotic assisted excision of a left ventricular myxoma. Interact Cardiovasc Thorac Surg.

